# Utilization of Maternal Health Care Among Immigrant Mothers in New York City, 2016–2018

**DOI:** 10.1007/s11524-021-00584-5

**Published:** 2021-11-22

**Authors:** Sheela Maru, Lily Glenn, Kizzi Belfon, Lauren Birnie, Diksha Brahmbhatt, Max Hadler, Teresa Janevic, Simone Reynolds

**Affiliations:** 1 Department of Health System Design and Global Health, Icahn School of Medicine at Mount Sinai, New York, NY USA; 2 Department of Obstetrics, Gynecology, and Reproductive Science, Icahn School of Medicine at Mount Sinai, New York, NY USA; 3 New York City Health + Hospitals/Elmhurst, New York, NY USA; 4 Center for Health Equity and Community Wellness, New York City Department of Health and Mental Hygiene, 42-09 28th Street, Long Island City, NY 11101 USA; 5 Division of Family and Child Health, New York City Department of Health and Mental Hygiene, Long Island City, NY USA; 6 Weill Cornell Medical College, New York, NY USA; 7 Independent Consultant, Brooklyn, NY USA; 8 Blavatnik Family Women’s Health Research Institute, Icahn School of Medicine at Mount Sinai, New York, NY USA; 9 Department of Population Health Science and Policy, Icahn School of Medicine at Mount Sinai, New York, NY USA; 10 Department of Epidemiology and Biostatistics, School of Public Health, SUNY Downstate Health Sciences University, Brooklyn, NY USA

## Abstract

Immigrant women represent half of New York City (NYC) births, and some immigrant groups have elevated risk for poor maternal health outcomes. Disparities in health care utilization across the maternity care spectrum may contribute to differential maternal health outcomes. Data on immigrant maternal health utilization are under-explored in the literature. We conducted a cross-sectional analysis of the population-based NYC Pregnancy Risk Assessment Monitoring System survey, using 2016–2018 data linked to birth certificate variables, to explore self-reported utilization of preconception, prenatal, and postpartum health care and potential explanatory pathways. We stratified results by maternal nativity and, for immigrants, by years living in the US; geographic region of origin; and country of origin income grouping. Among immigrant women, 43% did not visit a health care provider in the year before pregnancy, compared to 27% of US-born women (risk difference [RD] = 0.16, 95% CI [0.13, 0.20]), 64% had no dental cleaning during pregnancy compared to 49% of US-born women (RD = 0.15, 95% CI [0.11, 0.18]), and 11% lost health insurance postpartum compared to 1% of US-born women (RD = 0.10, 95% CI [0.08, 0.11]). The largest disparities were among recent arrivals to the US and immigrants from countries in Central America, South America, South Asia, and sub-Saharan Africa. Utilization differences were partially explained by insurance type, paternal nativity, maternal education, and race and ethnicity. Disparities may be reduced by collaborating with community-based organizations in immigrant communities on strategies to improve utilization and by expanding health care access and eligibility for public health insurance coverage before and after pregnancy.

**Supplementary Information:**

The online version contains supplementary material available at 10.1007/s11524-021-00584-5.

## Introduction

The United States (US) is home to the largest population of immigrants in the world, the majority of who live in just 20 major metropolitan areas [1]. The immigrant population in New York City (NYC) is large and diverse, composed primarily of immigrants from Latin America, Asia, and the Caribbean [2]. NYC immigrants speak over 200 languages and account for 36% of the city’s population, or 3 million people [2]. In 2018, half of all births in NYC were to women^1^ born outside the US [3]. Some immigrant women have poorer maternal health outcomes, compared to US-born women, including higher rates of gestational diabetes [4] and severe maternal morbidity [5, 6]. Disparities in maternal health care utilization may contribute to these outcomes, as suggested by the existing literature, though much of these data are from Europe, and US immigrant maternal health care utilization is underexplored [7].

Access to and utilization of health care across the maternity care spectrum—preconception, prenatal, and postpartum—is important for optimal maternal health. Preconception care is essential for managing chronic conditions that are risk factors for severe maternal morbidity [8]. Timely and adequate prenatal care is the most common recommendation for improving maternal health outcomes [9]. Postpartum care, in the “4th trimester,” is important for optimizing long-term maternal health [10]. Compared to US-born women, immigrant women are less likely to have a usual source of care [11], and more likely to have inadequate and delayed initiation of prenatal care [12, 13].

Immigrant women may have poorer access to maternal health care due to lack of insurance. Health insurance contributes to health care utilization, and disparities in health insurance status by nativity are well documented [14–17]. Exclusionary health coverage policies, such as Medicaid ineligibility for some immigrant groups, also limit access to care [18]. In NYC, nearly 18% of immigrants are uninsured compared to 6% of the US-born population [19].

Additional factors may contribute to lower utilization of health care services among immigrants, including the complex health care system and lack of awareness of available services [20], cultural and language discordance in health care provision [21], and anti-immigrant rhetoric and policies, such as the Trump administration’s expanded definition of the public charge rule [22]. Health care utilization among immigrants may also differ based on time in the US, with more time in the US associated with better access to care [23, 24]. Furthermore, research suggests that utilization patterns may vary by country or region of origin, with European immigrants having better rates of utilization than other immigrants [25].

The maternal health service utilization literature contains several gaps. Maternal health service utilization in the preconception period is under-explored, and no literature to date has examined utilization of postpartum services among immigrant populations in NYC. Additionally, most studies have broadly categorized immigrant populations, which likely masks differences within pan-ethnic populations. Understanding immigrant health service utilization patterns by other categorizations, such as region of origin and country of origin income grouping as defined by the World Bank, may elucidate mechanisms of maternal health disparities and highlight immigrant communities with utilization gaps, which may or may not be geographically clustered. Addressing health care underutilization has implications for reducing severe maternal morbidity and mortality and improving maternal health across the life course.

**Fig. 1:**
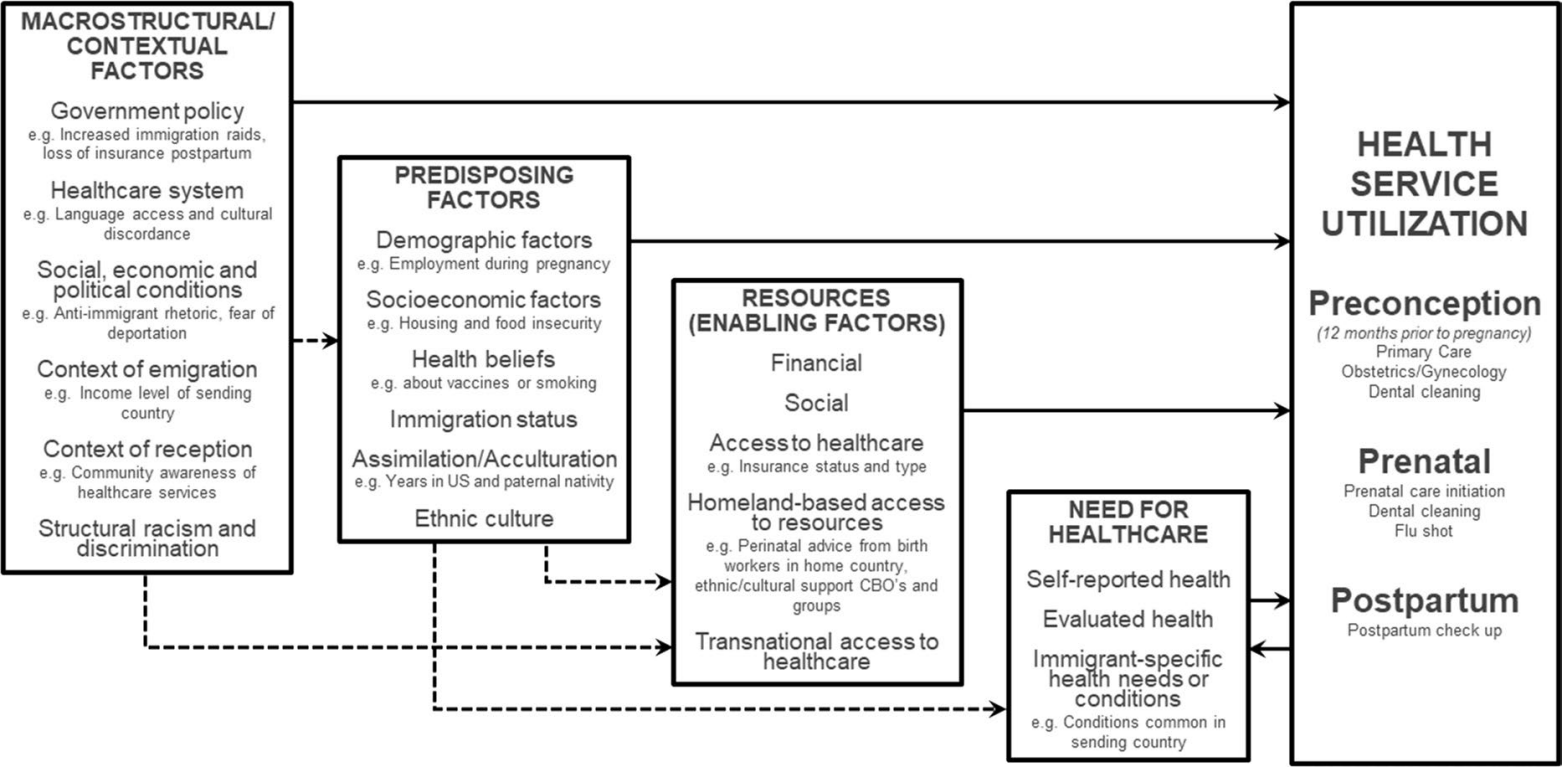
An analytical framework for maternal health service utilization among immigrant women in NYC. A solid line denotes a direct effect; a broken line indicates that some of the factors within the category have an indirect effect on maternal health service utilization

In this study, we fill gaps in the literature by examining data from a survey of women who gave birth in NYC from 2016 to 2018, focusing on utilization of preconception, prenatal, and postpartum care by nativity, and for immigrant women, by years in the US, region of origin, and country of origin income grouping. We further seek to explore the factors that have the most impact on these utilization outcomes across the maternity spectrum. Focusing on NYC allows for an examination of utilization patterns across multiple immigrant populations while holding constant the overall socio-cultural, political, and economic context. To guide our analyses, we adapted Yang et al.’s analytical framework for immigrant health service utilization [26]. Our framework (Fig. 1) details the macrostructural/contextual, predisposing, enabling, and health need factors specific to immigrant women in NYC.

### Community Engagement

The study was guided by input from community stakeholders who work with immigrant women in NYC, to better interpret the data and increase utility for community advocacy. Across a series of meetings in each stage of the research process, stakeholders provided feedback on and refined actionable research questions, helped to contextualize results of analyses, and identified avenues for dissemination to policymakers and community members, such as holding community conversations featuring research results tailored to specific immigrant groups.

## Methods

This study emerged from Health Data for New York City (HD4NYC), a partnership between the New York Academy of Medicine and the NYC Department of Health and Mental Hygiene (NYC Health Department). HD4NYC promotes policy-relevant population health research to improve health equity in NYC and emphasizes community engagement throughout the research process.

### Data Sources

We conducted a cross-sectional analysis of data from the 2016–2018 NYC Pregnancy Risk Assessment Monitoring System (NYC PRAMS), a population-based surveillance system that collects self-reported data on maternal experiences and behaviors before, during, and shortly after pregnancy, linked with select variables from the birth certificate. The NYC Health Department administered the NYC PRAMS survey in coordination with the Centers for Disease Control and Prevention. The NYC PRAMS survey team drew a stratified random sample without replacement of NYC residents with a live birth from birth certificate data at 2 to 4 months postpartum. Sampled women were sent up to three mail surveys in English, Spanish, or Chinese and, if non-responsive, were followed up with via telephone. NYC PRAMS data were weighted to account for complex survey design, non-response, and non-coverage, to be representative of all NYC residents with a live birth. Non-response weights were calculated using variables related to timing of prenatal care initiation, delivery hospital, and maternal demographic characteristics, including education, age, marital status, race, ethnicity, insurance type, WIC participation, parity, and county of residence. Maternal nativity was not utilized in the calculation of NYC PRAMS survey weights. Annual weighted response rates ranged from 65 to 72%. The NYC Health Department Institutional Review Board reviewed this project and determined it had “exempt” status (IRB no. 19–112).

### Measures

The primary exposure of interest was self-reported maternal nativity (US-born vs. immigrant) on the infant’s birth certificate. We categorized immigrant women by years living in the US (0–4, 5–9, or 10 +); World Bank geographic region of origin [27] (Europe and Central Asia, Middle East and North Africa, sub-Saharan Africa, South Asia, East Asia and Pacific, or Latin America and the Caribbean, which we further disaggregated to Caribbean, Central America, or South America using sub-region categories standardized by the United Nations) [28]; and 2018 World Bank income group for country of origin (low-, lower-middle-, upper-middle-, or high-income economies) [27]. The sample size precluded us from categorizing immigrants by individual countries.

We dichotomized maternal health care utilization during the preconception, prenatal, and postpartum periods as reported on the PRAMS questionnaire. Preconception outcomes were no health care visits in the 12 months before pregnancy overall and by visit type (routine checkup with OB/GYN, routine checkup with family doctor, primary care visit (OB/GYN or family doctor), and for dental cleaning). Prenatal outcomes were no or late (third trimester) prenatal care, delayed initiation (second or third trimester) of prenatal care, no flu shot during the 12 months prior to giving birth, and no dental cleaning during pregnancy. Postpartum outcomes were no postpartum check-up and loss of health insurance coverage in the postpartum period.

Covariates were selected to reflect our analytical framework (Fig. 1) as potential explanatory pathways to maternal health care utilization, and by availability in the data sources. Covariates included insurance type during the preconception, prenatal, and postpartum periods (private, Medicaid or other public, other, none, or unknown); race and ethnicity (Latina, White non-Latina, Black non-Latina, Asian or Pacific Islander, or multiracial or other race); maternal education (less than high school, high school/GED, some college/associates degree, or bachelor degree or higher); maternal age (< 20, 20–29, 30–39, or ≥ 40) and parity (primiparous vs. multiparous); comorbidities 3 months before pregnancy (body mass index (underweight, normal, overweight, or obese), hypertension (yes/no), and diabetes (yes/no)); paternal nativity (US-born, immigrant, or unknown); and experiences of racial bias in the 12 months before giving birth (yes/no). All covariates came from the birth certificate, except for insurance type, pre-pregnancy health status, and experiences of racial bias, which were collected via NYC PRAMS.

We explored missing data for all covariates and found that missing data were more common for prenatal insurance type (7% missing) and paternal nativity status (10% missing). All other covariates had less than 4% missing data. To determine whether data were missing at random for insurance type and paternal nativity, we created cross-tabulations of the missing and non-missing responses by maternal nativity, years in the US, and World Bank geographic regions. Immigrant respondents were disproportionately represented in the missing insurance data across the maternal health spectrum (82% of the missing data preconception insurance was among immigrants, 79% for prenatal insurance, and 85% for postpartum insurance), so we added an “unknown” category for insurance type to retain those non-respondents in the regression models. Immigrants tend to be a significant portion of individuals who are uninsured or have transient insurance in the NYC population, so the “unknown” category is a marker for this risk group. Although maternal nativity, years in US, and geographic regions were similarly represented across missing and non-missing responses for paternal nativity, we created an “unknown” category for that covariate because missing birth certificate data on the father can have conceptual meaning regarding paternal involvement. Specifically, evidence has shown that infants with underreported paternal nativity tend to have poorer birth outcomes compared to their counterparts [29, 30].

### Analysis

We calculated weighted prevalence estimates and 95% confidence intervals for all baseline characteristics, covariates, and outcomes of interest. We used chi-square tests to test associations between maternal nativity and baseline demographic, socioeconomic, and macrostructural characteristics. We examined bivariate associations between maternal nativity and health care utilization using predicted marginal effects from logistic regression to calculate crude risk differences (RDs). Unadjusted RDs were calculated for immigrant women overall compared to US-born women, and stratified by years in the US, World Bank geographic regions, and World Bank income groups. Adjusted risk differences were calculated from predicted marginals in the logistic regression model, including covariates individually and combined. We did not conduct a formal causal mediation analysis, as this study is exploratory. Analyses were conducted using SAS-callable SUDAAN in SAS Enterprise Guide 7.1 to account for complex survey design, non-response, and non-coverage, enabling the generalizability of findings to all NYC resident births during 2016–2018. We present the results first with an emphasis on the unadjusted risk differences, and then, as an extension of our exploratory approach, the adjusted risk differences with covariates included.

## Results

We compared a variety of demographic, socioeconomic, and macrostructural characteristics by nativity (Table 1). Race and ethnicity differed by nativity, as immigrant women were primarily Latina and Asian or Pacific Islanders. Immigrant women had lower education levels, compared to US-born women, and higher proportions of being unemployed, uninsured, in poverty, participating in WIC, and experiencing food insecurity. Immigrant women were also more likely than US-born women to report experiencing racial discrimination in the year before birth and to have an immigrant father of the infant listed on the birth certificate.

**Table 1:** PRAMS sample characteristics by nativity, 2016–2018

Characteristic	US-born			Immigrant			*P*-value
	Unweighted *N*	Weighted *N*	% (95% CI)	Unweighted *N*	Weighted *N*	% (95% CI)	
**Total sample**	2018	151,200	47.5 (45.8, 49.2)	2249	167,095	52.5 (50.8, 54.2)	–
**Years in US**^a^							
0–4 years	–	–	–	619	47,000	28.4 (26.3, 30.6)	
5–9 years	–	–	–	553	42,732	25.8 (23.8, 27.9)	
10 + years	–	–	–	1047	75,760	45.8 (43.5, 48.1)	
**Region of origin**^a,i^							–
Caribbean	–	–	–	562	36,489	22.1 (20.3, 24.1)	
Central America	–	–	–	292	22,995	14.0 (12.4, 15.7)	
South America	–	–	–	285	21,182	12.9 (11.4, 14.5)	
Europe — Central Asia	–	–	–	287	22,947	13.9 (12.4, 15.6)	
Middle East — North Africa	–	–	–	91	6255	3.8 (3.0, 4.8)	
Sub-Saharan Africa	–	–	–	136	10,117	6.1 (5.1, 7.4)	
South Asia	–	–	–	227	17,127	10.4 (9.0, 11.9)	
East Asia — Pacific	–	–	–	338	27,636	16.8 (15.1, 18.6)	
**Country of origin income group**^a^							–
Low Income	–	–	–	185	11,623	7.0 (5.8, 8.3)	
Lower-Middle Income	–	–	–	476	36,314	21.7 (19.9, 23.7)	
Upper-Middle Income	–	–	–	1298	98,659	59.1 (56.8, 61.3)	
High Income	–	–	–	288	20,382	12.2 (10.8, 13.8)	
**Race and ethnicity**^a^							< 0.001
Latina	451	34,134	22.6 (20.6, 24.7)	735	58,167	34.8 (32.6, 37.1)	
White non-Latina	932	73,124	48.4 (46.0, 50.8)	401	31,412	18.8 (17.1, 20.7)	
Black non-Latina	508	35,166	23.3 (21.2, 25.5)	481	28,215	16.9 (15.2, 18.7)	
Asian or Pacific Islander	91	6584	4.4 (3.5, 5.4)	594	47,044	28.2 (26.1, 30.3)	
Multiracial or other race	34	2060	1.4^b^ (0.9, 2.0)	36	2204	1.3^b^ (0.9, 1.9)	
**Racial discrimination**^a,e^							0.01
Experienced	135	9741	6.7 (5.6, 8.1)	208	14,771	9.2 (7.9, 10.6)	
Not experienced	1797	135,011	93.3 (91.9, 94.4)	1958	146,564	90.8 (89.4, 92.1)	
**Language of survey admin**							< 0.001
English	1970	147,411	97.5 (96.6, 98.1)	1525	108,752	65.1 (62.8, 67.3)	
Other (Spanish or Chinese)	48	3790	2.5^b^ (1.9, 3.4)	724	58,343	34.9 (32.7, 37.2)	
**Age, years**							0.078
< 20	68	5490	3.6 (2.8, 4.7)	37	3464	2.1^b^ (1.5, 2.9)	
20–29	762	61,140	40.4 (38.1, 42.9)	846	66,919	40.0 (37.8, 42.4)	
30–39	1061	75,866	50.2 (47.8, 52.6)	1211	86,930	52.0 (49.7, 54.3)	
40 +	127	8704	5.8 (4.8, 7.0)	155	9782	5.9 (4.9, 7.0)	
**Education**^a^							< 0.001
Less than high school	196	16,330	10.8 (9.4, 12.5)	492	39,688	23.8 (21.8, 25.8)	
High school grad/GED	399	31,743	21.0 (19.1, 23.1)	530	39,426	23.6 (21.7, 25.7)	
Some college/assoc. deg.	498	36,481	24.2 (22.2, 26.3)	486	34,230	20.5 (18.7, 22.4)	
Bachelor’s deg. or higher	920	66,285	43.9 (41.6, 46.3)	738	53,660	32.1 (30.0, 34.3)	
**Employment during pregnancy**^a^							< 0.001
Employed	1448	107,304	71.0 (68.8, 73.2)	1046	76,209	45.7 (43.4, 48.0)	
Unemployed	568	43,763	29.0 (26.8, 31.2)	1197	90,677	54.3 (52.0, 56.6)	
**Parity**^a^							< 0.001
First birth	950	69,809	46.2 (43.8, 48.6)	911	65,312	39.1 (36.9, 41.4)	
Previous birth	1067	81,358	53.8 (51.4, 56.2)	1336	101,736	60.9 (58.6, 63.1)	
**Household poverty**^a,c^							< 0.001
≤ 100% FPL	431	34,155	27.1 (24.7, 29.5)	794	61,083	46.7 (44.1, 49.4)	
101–200% FPL	283	21,115	16.7 (14.8, 18.8)	398	28,463	21.8 (19.7, 24.0)	
> 200% FPL	999	70,954	56.2 (53.6, 58.8)	568	41,139	31.5 (29.1, 33.9)	
**Food insecurity**^a,d^							< 0.001
Concerns about food	178	13,210	9.2 (7.9, 10.8)	442	31,980	20.2 (18.3, 22.2)	
No concerns about food	1742	129,713	90.8 (89.2, 92.1)	1684	126,534	79.8 (77.8, 81.7)	
**WIC particip. during pregnancy**^a^							< 0.001
Yes	759	58,509	38.8 (36.5, 41.2)	1266	96,379	57.9 (55.6, 60.2)	
No	1252	92,226	61.2 (58.8, 63.5)	974	70,084	42.1 (39.8, 44.4)	
**Paternal nativity**							< 0.001
US-born	1409	107,167	70.9 (68.6, 73.0)	316	21,795	13.0 (11.6, 14.6)	
Immigrant	408	28,776	19.0 (17.2, 21.0)	1725	129,690	77.6 (75.6, 79.5)	
Unknown	201	15,257	10.1 (8.7, 11.7)	208	15,609	9.3 (8.1, 10.8)	
**Insurance type — preconception**							< 0.001
Private	1288	93,383	61.8 (59.3, 64.1)	882	62,425	37.4 (35.2, 39.6)	
Medicaid/other pub. ins.^f^	613	49,062	32.4 (30.2, 34.8)	731	56,068	33.6 (31.4, 35.8)	
Other insurance	51	3739	2.5^b^ (1.8, 3.4)	172	12,747	7.6 (6.5, 9.0)	
Uninsured	48	3607	2.4^b^ (1.7, 3.3)	378	29,334	17.6 (15.9, 19.4)	
Unknown^g^	Suppressed^h^			86	6521	3.9 (3.1, 4.9)	
**Insurance type — prenatal**							< 0.001
Private	1220	87,727	58.0 (55.6, 60.4)	823	57,946	34.7 (32.5, 36.9)	
Medicaid/other pub. ins.^f^	672	53,969	35.7 (33.4, 38.1)	990	75,721	45.3 (43.0, 47.6)	
Other insurance	55	3997	2.6^b^ (1.9, 3.6)	168	12,687	7.6 (6.5, 8.9)	
Uninsured	Suppressed^h^			Suppressed^h^			
Unknown^g^	67	5215	3.4 (2.6, 4.5)	247	19,351	11.6 (10.2, 13.2)	
**Insurance type — postpartum**							< 0.001
Private	1178	84,511	55.9 (53.5, 58.3)	803	56,526	33.8 (31.7, 36.0)	
Medicaid/other pub. ins.^f^	729	58,112	38.4 (36.1, 40.8)	903	68,319	40.9 (38.6, 43.2)	
Other insurance	78	5747	3.8 (3.0, 4.9)	233	18,632	11.2 (9.8, 12.7)	
Uninsured	Suppressed^h^			233	17,787	10.6 (9.3, 12.2)	
Unknown^g^	Suppressed^h^			77	5831	3.5 (2.7, 4.4)	

^a^Sample size does not total the sample size overall or by nativity, as some respondents did not answer question

^b^Interpret with caution due to small unweighted sample size (< 60)

^c^Federal poverty levels (FPL) calculated using Health and Human Services poverty guidelines, 2016–2018

^d^In the last 30 days

^e^In the year before birth

^f^Other public insurance includes Child Health Plus, Family Health Plus, and Family Planning Benefit Program

^g^Unknown insurance includes missing and inconsistent responses

^h^Results suppressed due to unweighted sample size < 33

^i^North American countries are excluded

### Preconception Outcomes

**Fig. 2:**
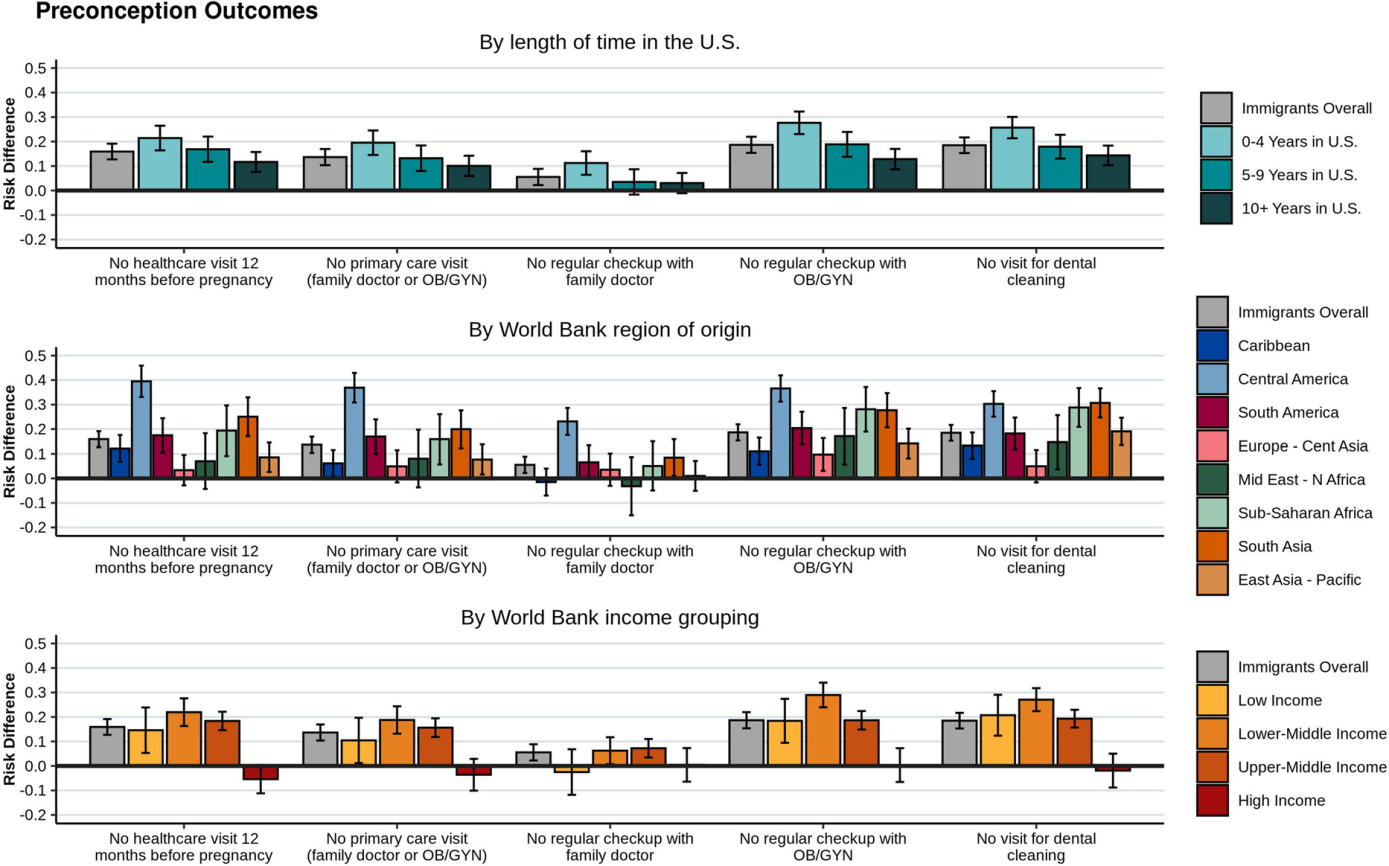
Preconception health care utilization outcomes for immigrant women giving birth in NYC, 2016–2018. Unadjusted risk differences and 95% confidence intervals, compared to US-born women, are shown for immigrants overall, and by length of time in the US, World Bank region of origin, and World Bank income grouping

Immigrants had lower utilization of preconception health care than US-born women (Fig. 2 for RDs and Supp. Table A1 for prevalence estimates). Notably, immigrant women had higher risk of not having a health care visit during the 12 months before pregnancy (RD = 0.16, 95% CI [0.13, 0.20]), not having a primary care visit (RD = 0.14, 95% CI [0.10, 0.17]) or regular visit with an OB/GYN (RD = 0.19, 95% CI [0.15, 0.22]), and not receiving a dental cleaning (RD = 0.18, 95% CI [0.15, 0.22]), compared to US-born women. Immigrant women living in the US for the shortest length of time had the highest risk differences, compared to US-born women, with differences in health care utilization decreasing as time spent in the US increased (Fig. 2). Stratified by geographic regions and compared to US-born women, women with the highest risk of no preconception primary care visit were from countries in Central America (RD = 0.37, 95% CI [0.31, 0.43]), South Asia (RD = 0.20, 95% CI [0.12, 0.28]), South America (RD = 0.17, 95% CI [0.10, 0.24]), and sub-Saharan Africa (RD = 0.16, 95% CI [0.06, 0.26]). This was consistent across all preconception health care utilization outcomes. Women from East Asian-Pacific countries were also at higher risk, compared to US-born women, for not having a dental visit (RD = 0.19, 95% CI [0.14, 0.25]) (Fig. 2). The smallest risk differences across most preconception outcomes, as compared to US-born women, were among women from countries in Europe and Central Asia. Stratified by income groups and compared to US-born women, immigrant women from lower-middle income countries had the largest utilization differences for most preconception outcomes.

### Prenatal Outcomes

**Fig. 3:**
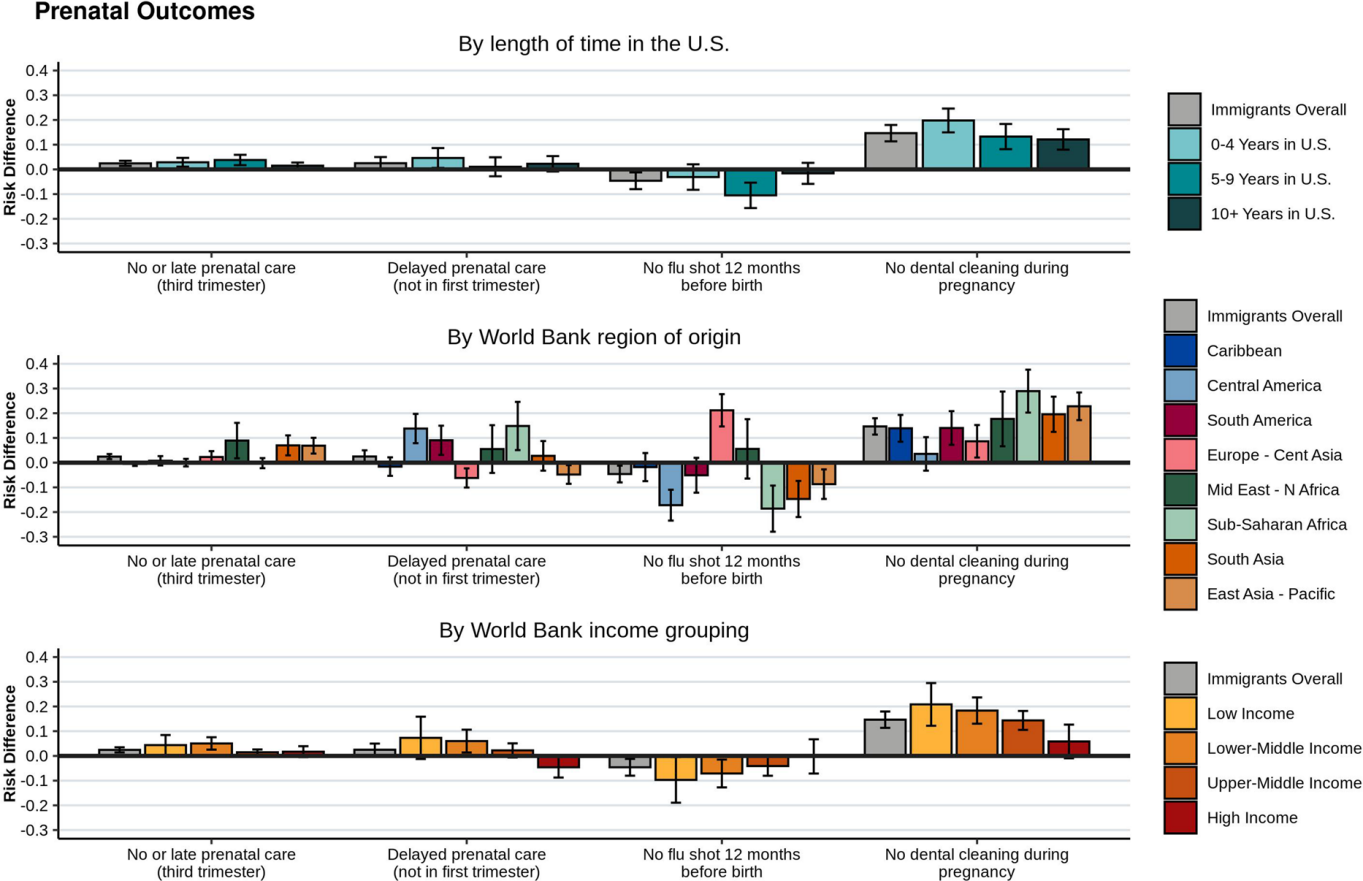
Prenatal health care utilization outcomes for immigrant women giving birth in NYC, 2016–2018. Unadjusted risk differences and 95% confidence intervals, compared to US-born women, are shown for immigrants overall, and by length of time in the US, World Bank region of origin, and World Bank income grouping

Immigrant women had lower utilization of most prenatal health care outcomes compared to US-born women (Fig. 3 for RDs and Supp. Table A2 for prevalence estimates). Immigrant women were at slightly higher risk than US-born women of not having a prenatal care visit or having a late one (i.e., visit occurred in the third trimester) (RD = 0.02, 95% CI [0.01, 0.03]) and having a delayed prenatal visit (i.e., first visit occurred after the first trimester) (RD = 0.02, 95% CI [0.00, 0.05]). Compared to US-born women, immigrant women were at higher risk of not having a dental cleaning during pregnancy (RD = 0.15, 95% CI [0.11, 0.18]), and the risk differences for no dental cleaning were slightly lower with more time in the US (Fig. 3). We observed no other distinct patterns relating to time in the US for the other prenatal outcomes. Immigrant women were less likely than US-born women to have not received a flu shot during the 12 months before birth (RD =  − 0.05, 95% CI [− 0.08, − 0.01]), with women in the US for 5–9 years being least likely to have not received a flu shot (RD =  − 0.11, 95% CI [− 0.16, − 0.05]).

When examined by geographic regions, risk of delayed prenatal care utilization was greatest among women from sub-Saharan African (RD = 0.15, 95% CI [0.05, 0.25]), Central American (RD = 0.14, 95% CI [0.08, 0.20]), and South American (RD = 0.09, 95% CI [0.03, 0.15]) countries, compared to US-born women. Women at smallest risk of not having a flu shot during the 12 months before birth, compared to US-born women, were from countries in sub-Saharan Africa (RD =  − 0.19, 95% CI [− 0.28, − 0.09] and Central America (RD =  − 0.17, 95% CI [− 0.23, − 0.11]), while the women at greatest risk of not having a flu shot were from countries in Europe and Central Asia (RD = 0.21, 95% CI [0.15, 0.28]). When stratified by income groups, immigrant women from low-income countries had the highest risk of not having a dental cleaning during pregnancy (RD = 0.21, 95% CI [0.12, 0.29]), compared to US-born women, with decreasing risk observed as country incomes increased.

### Postpartum Outcomes

**Fig. 4:**
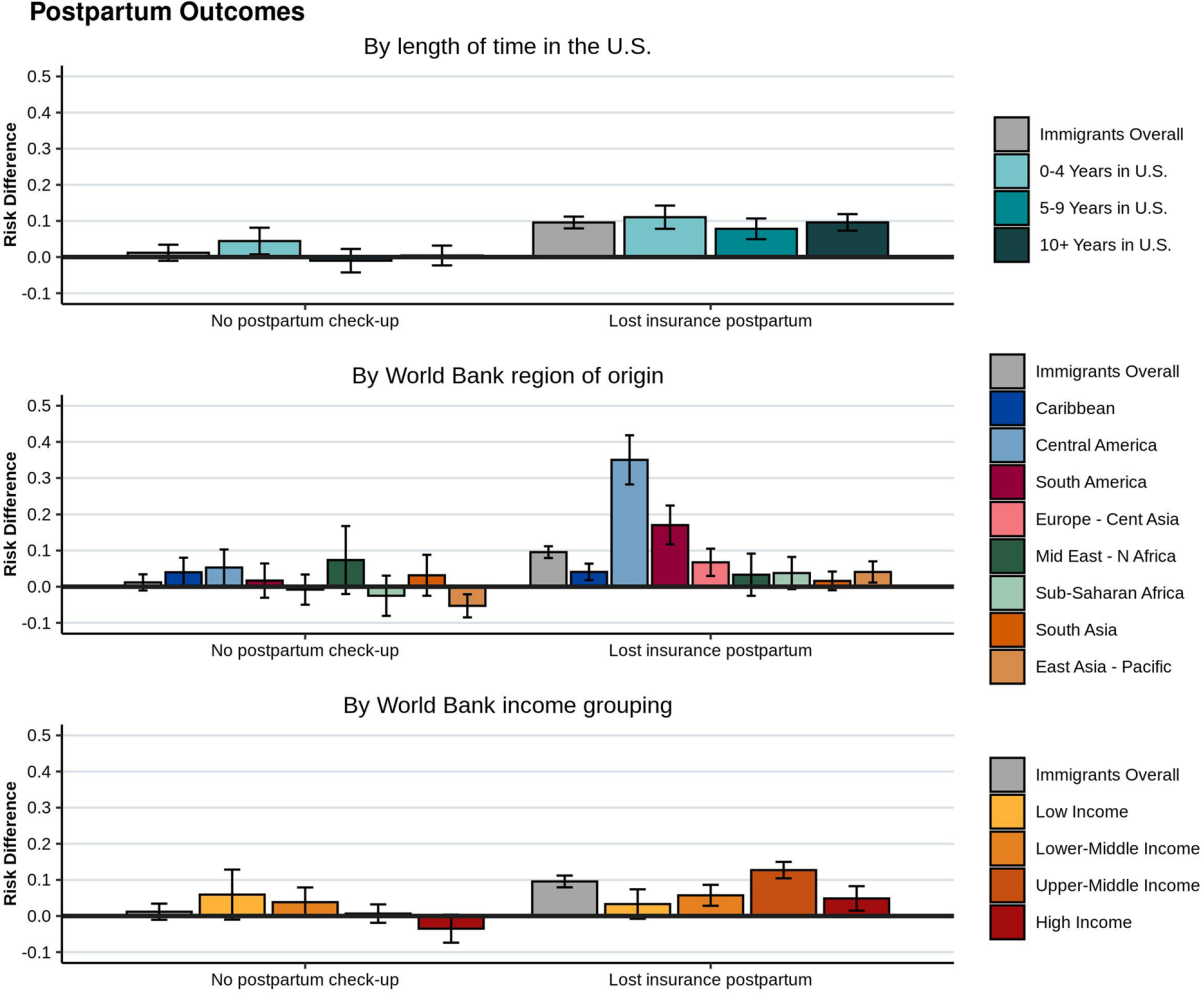
Postpartum health care utilization outcomes for immigrant women giving birth in NYC, 2016–2018. Unadjusted risk differences and 95% confidence intervals, compared to US-born women, are shown for immigrants overall, and by length of time in the US, World Bank region of origin, and World Bank income grouping

Immigrant women had higher risk of losing insurance during the postpartum period (RD = 0.10, 95% CI [0.08, 0.11]), compared to US-born women. This did not differ based on time in the US (Fig. 4 for RDs and Supp. Table A3 for prevalence estimates). Among all women who lost insurance postpartum (weighted *N* = 16,596), 55% had no preconception insurance. This was compared to all women who maintained their insurance postpartum (weighted *N* = 271,976), where only 7% had no preconception insurance. The vast majority (92%) of women who lost insurance postpartum were immigrants. Postpartum visit attendance was similar between immigrant and US-born women (RD = 0.01, 95% CI [− 0.1, 0.03]). However, when categorized by time spent in the US, immigrant women in the US for 0–4 years had somewhat greater risk of not having a postpartum checkup (RD = 0.04, 95% CI [0.01, 0.08]), compared to US-born women.

When categorized by geographic region, immigrant women from Central American (RD = 0.35, 95% CI [0.28, 0.42]) and South American countries (RD = 0.17, 95% CI [0.12, 0.22]) had the highest risk of losing insurance postpartum, compared to US-born women (Fig. 4). Immigrant women from Central American countries had a slightly higher risk of not having a postpartum appointment (RD = 0.05, 95% CI [0.00, 0.10]), compared to US-born women, while immigrant women from East Asian-Pacific countries had lower risk (RD =  − 0.05, 95% CI [− 0.08, − 0.02]). Stratified by income group, women from upper-middle income countries had greatest risk of losing insurance postpartum (RD = 0.13, 95% CI [0.10, 0.15]), while women from low-income countries had the lowest risk (RD = 0.03, 95% CI [− 0.01, 0.07]), compared to US-born women.

We explored other insurance type trajectories across maternal health periods. From the preconception to prenatal period and from the prenatal to postpartum period, 7% of respondents overall moved from private insurance to Medicaid or other public insurance and about 3% moved from Medicaid or other public insurance to private insurance. Differences between immigrant and US-born women were minor or non-existent.

### Potential Explanatory Factors

For most preconception outcomes, adjusting separately for insurance type, paternal nativity, maternal education, and race and ethnicity reduced the RDs between US-born and immigrant mothers, suggesting that these covariates may contribute to health care utilization differences (Supp. Table A4). We observed lowest utilization of preconception health services among mothers with no insurance, those with less than a high school education, those identifying as Latina, and where the infant’s father was an immigrant or paternal nativity was unknown (results not shown). For most prenatal and postpartum outcomes, adjusting for insurance type, paternal nativity, maternal education, and race and ethnicity led to small reductions in the risk differences. Adjustments for age, parity, comorbidities, and experience of racial discrimination had minimal impact on risk differences across preconception, prenatal, and postpartum outcomes.

Table 2 details the adjusted risk differences between US-born and immigrant mothers, including all covariates. Accounting for all covariates in the regression model reduced, but did not eliminate, risk differences for nearly all outcomes.

**Table 2:** Unadjusted and fully adjusted risk differences, immigrants compared to US-born (ref)

Outcome	Unadjusted		Adjusted for covariates^a^	
	RD	95% CI	RD	95% CI
**Preconception**				
No healthcare visit 12 months before pregnancy	0.16	0.13, 0.19	0.03	0.01, 0.08
No primary care visit (either fam doc or OB/GYN)	0.14	0.10, 0.17	0.02	− 0.02, 0.06
No regular checkup with family doctor	0.06	0.02, 0.09	− 0.00	− 0.05, 0.04
No regular checkup with OB/GYN	0.19	0.15, 0.22	0.06	0.02, 0.11
No visit for dental cleaning	0.18	0.15, 0.22	0.05	0.01, 0.09
**Prenatal**				
No or late prenatal care (third trimester)	0.02	0.01, 0.03	0.01	− 0.00, 0.02
Delayed prenatal care (not in first trimester)	0.02	0.00, 0.05	− 0.01	− 0.04, 0.03
No flu shot 12 months before birth	− 0.05	− 0.08, − 0.01	− 0.01	− 0.06, 0.03
No dental cleaning during pregnancy	0.15	0.11, 0.18	0.09	0.04, 0.13
**Postpartum**				
No postpartum check-up	0.01	− 0.01, 0.03	− 0.03	− 0.06, − 0.01
Lost insurance postpartum	0.10	0.08, 0.11	0.07	0.05, 0.09

^a^ “Lost insurance postpartum” outcome is adjusted for maternal education, race and ethnicity, age, parity, comorbidities three months before pregnancy (diabetes, hypertension, and BMI), experience of racial discrimination, and paternal nativity. All other outcomes are adjusted for the previously listed covariates plus insurance type

## Discussion

We found sizeable differences in the utilization of maternal health care between immigrant and US-born women in NYC. These differences were most prominent among recently arrived immigrant women, and those from Central America, South Asia, sub-Saharan Africa, and South America (listed in descending order of RDs, compared to US-born women, for some but not all outcomes). Immigrant women from high-income countries had similar utilization as US-born women. Notably, we reported large differences in preconception care and postpartum insurance loss, points of care previously under-explored in immigrant health literature. Adjusting for insurance type, paternal nativity, maternal education, and race and ethnicity reduced some risk differences comparing immigrants and US-born women, suggesting that some of the potential explanatory mechanisms considered here may warrant further investigation.

Our study builds on previous research highlighting differences in prenatal care utilization between immigrant and US-born women. Late initiation of prenatal care is the most commonly studied utilization measure, and most studies found immigrant women to be at greater risk [31, 32]. We broadened the scope of utilization and showed large differences in preconception care and postpartum insurance loss, together suggesting limited access to non-pregnancy care. Obstetricians and public health leaders increasingly recognize preconception and postpartum periods as crucial to optimizing maternal and infant health outcomes, in addition to long-term maternal health. For example, the preconception period is important for preventing severe maternal morbidity by screening for and managing pre-existing conditions [8]. Regular access to care outside of pregnancy also improves access to family planning, allowing for well-informed fertility decisions. Not having health care in the preconception period may lead to unintended pregnancy, which is a strong risk factor for adverse birth outcomes [33]. Finally, non-pregnant periods are an opportunity for improving health literacy and risk awareness; referral to mental health services, smoking cessation, or other substance use services; and promoting a healthy lifestyle [34]. Our findings suggest that immigrant women are less likely to receive this care, compared to US-born women.

A key contribution of our study is our use of country of origin to categorize immigrant women, centering our analysis on shared characteristics such as circumstances of immigration and culture. The standard approach based on the Office of Management and Budget is to categorize race and ethnicity as Hispanic or Latina, non-Hispanic Black, non-Hispanic White, and non-Hispanic Asian, and researchers often examine immigrant vs. US-born outcomes within those groups. Our results underscore how the standard approach can obscure important differences. For example, compared to US-born women, Central Americans had by far the greatest risk of losing insurance postpartum (RD = 0.35, 95% CI [0.28, 0.42]), and South Asians had greater risk than East Asians of lacking a preconception health visit (RD = 0.25, 95% CI [0.17, 0.33] vs. RD = 0.09, 95% CI [0.03, 0.15]). We also used World Bank income categories to further inform the influence of country-of-origin context. For example, immigrant women from low or lower-middle income countries had the largest differences for most utilization outcomes. These findings provide evidence to advocate for interventions that are tailored to immigrant sub-populations.

We found a suggestion that insurance type was associated with differences in maternal health care utilization. We understand insurance type as a macrostructural factor directly impacting health service utilization as well as a predisposing factor and resource for immigrant women (Fig. 1). In NYC, Medicaid or other public insurance covers nearly half of immigrant women during pregnancy. Undocumented women can enroll in Medicaid when they are pregnant, which gives them insurance access up to 60 days after the end of pregnancy. However, they cannot access Medicaid outside of pregnancy, likely explaining the greater preconception and postpartum utilization disparities. Still, some immigrant groups had higher risk than US-born women of late or no prenatal care. One potential reason is that women who have health insurance in the preconception period may be more likely to have established medical providers, and thus can quickly access care when they discover they are pregnant. For example, researchers found that after Medicaid expansion in Ohio under the Affordable Care Act, pregnant women were more likely to be enrolled in Medicaid prior to pregnancy, and more likely to receive recommended elements of prenatal care [35]. Thus, our finding that immigrant women lack preconception care may influence both health status in pregnancy and the receipt of quality prenatal care, both of which impact health outcomes.

Our data revealed years in the US and paternal nativity as salient to utilization disparities. More recently arrived immigrants had the largest crude disparities, compared to US-born women, in nearly all the utilization outcomes examined. We also found a suggestion that paternal nativity was associated with some utilization outcomes. Both years in the US and paternal nativity are sometimes considered proxies of acculturation. Acculturation describes the process by which individuals adopt the attitudes, values, customs, beliefs, and behaviors of the host country [36]. Time in the US is often studied as a proxy for acculturation, with the assumption that the longer an immigrant lives in the receiving country, the more likely they are to assume its lifestyle and behavioral norms. We consider father’s nativity status as a proxy for acculturation due to its potential influence on maternal health behavioral norms. Pregnant immigrants whose partners are US-born fathers are more likely to have health behaviors like US-born mothers, including breastfeeding and smoking. Other mechanisms by which US-born fathers may influence maternal health care utilization include resources and knowledge needed to access the US health system, either through the father himself or his family or social network [37]. However, proxies for acculturation such as years in the US do not capture the “cultural” elements that may be underlying health, nor the macrostructural determinants of immigrant health such as racism, segregation, and exclusionary policies [38]. Years in the US and paternal nativity may reflect macrostructural factors, such as immigration law and Medicaid policy as much as acculturation (Fig. 1). For example, time in the US and paternal nativity may also be correlated with an immigration status considered “non-qualified” for federal assistance, including undocumented or unauthorized immigrants and immigrants with temporary visas [39]. Among immigrants with this status, fear of consequences on one’s status may discourage accessing care, even care to which they have a right, like pregnancy care in New York.

Adjusting for maternal education partially reduced some utilization disparities. Associations between education and prenatal care utilization are well-established [40]. Education serves as a proxy for socioeconomic status and health knowledge and beliefs [41]. Education also can serve as a proxy for circumstances of migration and reflect early life socioeconomic status in the country of origin. Further, education may reflect current transnational social networks in the country of origin and NYC, which may influence access to knowledge and resources [42]. Culturally tailored interventions should be inclusive of immigrants with less education. Improving access to education in immigrant communities may also represent a point of intervention itself, by increasing socioeconomic opportunity.

Adjusting for race and ethnicity partially attenuated differences in preconception healthcare utilization differences. Race and ethnicity, when interpreted as a social construct, reflects structural and interpersonal racism, which are actionable pathways [43, 44]. Policies to dismantle racism are vital to achieving immigrant health equity. However, race and ethnicity does not entirely account for immigrant utilization differences, suggesting anti-racism policies must be inclusive of immigrants.

### Policy Implications

Our findings of immigrant maternal care utilization disparities inform current policy debates regarding maternal morbidity and mortality, immigration, and Medicaid eligibility. The crisis of high US maternal mortality, particularly among Black women, is in the national spotlight. Federal legislation aims to improve maternal health outcomes and reduce racial and ethnic disparities. The American Rescue Plan Act gives states the option to temporarily extend Medicaid eligibility to 12 months postpartum but does not extend eligibility to immigrants currently ineligible for Medicaid [45]. In New York, this includes unauthorized immigrants, but depending on the state may also include legal permanent residents. Our findings highlight that immigrant women are at risk of missing postpartum care, and undocumented women should be fully included in any extension of post-pregnancy coverage. Our findings of disparities in preconception care also inform concurrent discussions around increasing public insurance eligibility that is not conditional on pregnancy. Strategies include removing the 5-year Medicaid waiting period for legal permanent residents, extending Medicaid to all low-income undocumented persons, and universal health coverage. There are approximately 3.5 million undocumented women of reproductive age in the US [46]. Given the sizeable population affected by current exclusions, such policy changes are important to reducing maternal health care utilization disparities.

Support linking immigrants to navigating the health care system, or moreover, simplification of the health care system may reduce disparities, particularly for recent immigrants who have among the lowest utilization rates in our study. These findings support programs for low-income immigrants that provide health navigation, awareness of health access rights, and information on immigration status and health insurance eligibility. The rapid change in immigration policy under the Trump administration and regulations such as the public charge rule have created additional layers of complexity and confusion [47, 48]. Their effect on immigrant’s health care will need to be explored in future studies.

### Limitations and Strengths

A notable limitation to our study is potential participation bias. Lawfully present and less-marginalized immigrant women may be more likely to participate, resulting in attenuation of apparent utilization disparities. Also, the survey was conducted in only three languages, likely resulting in the exclusion of women facing health care access issues due to language, again underestimating disparities. Furthermore, we had little information on mechanisms such as person-centered care, health care discrimination, health knowledge and beliefs, and documentation status. These limitations point to the importance of community-based qualitative research to elucidate barriers to care. Other limitations include cross-sectional study design and insufficient data to explore all countries of origin individually. Lastly, we did not conduct a formal causal mediation analysis, so caution is required when interpreting the adjusted analyses.

Despite these limitations, our research has notable strengths. We obtained time in the US and paternal nativity from the NYC birth certificate, unique data often unavailable in studies of immigrant perinatal health. We used novel categories of immigrants, including socioeconomic context of sending country, which are relevant to health disparities research and immigration policy. Finally, we engaged community stakeholders in the development and interpretation of our findings.

## Conclusion

Our study highlights disparities in maternal health care utilization among immigrants giving birth in NYC, with potential explanatory factors of insurance, paternal nativity, maternal education, and race and ethnicity. The most pronounced disparities were during the preconception and postpartum periods, both of which have become increasingly recognized as important in preventing severe maternal morbidity and mortality. Our analysis provides insights into the utilization patterns for specific ethnic sub-populations, which is a strength of data gathered from the diverse population of NYC. We encourage working closely with community-based organizations to identify tailored strategies for improving utilization. Future studies should further explore the reasons for disparities as well as test community-specific interventions to address these disparities. The policy implications of these data include expanding health care access prior to and beyond pregnancy, for which universal health insurance coverage would be a key strategy.

## Supplementary Information

Below is the link to the electronic supplementary material.

Supplementary file1 (XLSX 14 kb)

Supplementary file2 (XLSX 13 kb)

Supplementary file3 (XLSX 13 kb)

Supplementary file4 (XLSX 12 kb)

## Footnotes

1 Due to limitations in data collection of all pregnancy-capable genders, we consider the terms “mother,” “woman,” and “maternal” to apply to any person who is pregnant or capable of being pregnant, or who has delivered a child. When citing published research, we use the terms in those publications.
